# Multi‐State Implementation of the Alzheimer's Association Dementia Care Coordination (DCC) Program

**DOI:** 10.1111/jgs.70475

**Published:** 2026-04-30

**Authors:** Nancy A. Hodgson, Sarra Latif, Sonia Talwar, Liming Huang, Kerry Finegan, Lauren Stratton, Sam Fazio, Monica Moreno

**Affiliations:** ^1^ University of Pennsylvania School of Nursing Philadelphia Pennsylvania USA; ^2^ Alzheimer's Association Chicago Illinois USA

**Keywords:** dementia care coordination, dementia caregiving, implementation science, program evaluation

## Abstract

**Background:**

The Alzheimer's Association partnered with health systems in Illinois, Greater Missouri, and Washington to implement and evaluate the Knight Family dementia care coordination (DCC) program, a novel telephone‐based care coordination service that bridged the gap between clinical care and community support. Healthcare providers were trained to systematically identify dementia caregivers and refer them to Alzheimer's Association care consultants who provided personalized education, resources, and support. This innovative model addressed critical care coordination gaps in dementia care delivery.

**Methods:**

A mixed‐methods process evaluation assessed DCC implementation and caregiver outcomes. Caregivers completed validated surveys at baseline and follow‐up, including the PROMIS Self‐Efficacy for Managing Emotions scale and the general self‐efficacy scale (GSE). Surveys also captured action plan uptake, resource utilization, and barriers to implementation. Health system employees completed surveys and structured interviews about program satisfaction and workflow integration. The Consolidated Framework for Implementation Research (CFIR) and the RE‐AIM framework (Reach, Effectiveness, Adoption, Implementation, and Maintenance) guided identification of barriers, facilitators, and implementation outcomes.

**Results:**

Caregiver self‐efficacy scores were mixed: approximately 44%–45% improved on the self‐efficacy scales at follow‐up, while a nearly equal proportion (42%–44%) worsened. Mean PROMIS scores showed no significant change (all *p* > 0.4), and mean GSE scores declined slightly but significantly at 3 months. Although improvements in self‐efficacy were not consistently observed, caregivers demonstrated high program engagement: 70% completed action steps, 80% utilized community resources, and 90% demonstrated comprehension of their individualized action plans. Health system employees reported high program satisfaction, though clinician engagement remained a barrier.

**Conclusions:**

This multi‐state evaluation demonstrates the feasibility of implementing a telephone‐based dementia care coordination program across diverse health systems and highlighted strong caregiver engagement with program resources. However, self‐efficacy outcomes did not show consistent improvement. Controlled trials are needed to determine which caregivers benefit most and whether more intensive or targeted interventions are required.

## Graphical abstract

Facilitators and Barriers to DCC Program Implementation.

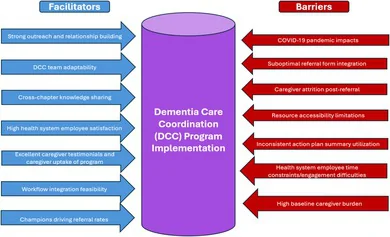

## 1 Introduction

Family care partners of persons living with dementia (PLWD) experience high levels of stress, burden, and limited access to supportive resources, despite evidence demonstrating the efficacy of caregiver interventions in improving emotional health and care quality [1, 2]. Traditional barriers including lack of awareness, time constraints, and fragmented care delivery limit caregiver service utilization despite availability.

The dementia care coordination (DCC) program served as a precursor to the Guiding an Improved Dementia Experience (GUIDE) model by addressing these traditional barriers through a systematic telephone‐based model that bridged clinical care and community support. Building on the successful Partners in Dementia Care (PDC) study conducted by the Department of Veterans Affairs (VA) and the Alzheimer's Association [3], DCC expanded this approach in three states by formally integrating referrals within clinical workflows. This model directly aligns with the ultimate goal of GUIDE—to connect families to services and supports. Following successful pilot testing in Massachusetts, DCC was implemented across the Greater Missouri, Washington, and Illinois chapters, providing timely evidence for scalable care coordination approaches as healthcare systems prepare for GUIDE implementation.

## 2 Methods

### 2.1 Program Description

The Alzheimer's Association partnered with health systems across three chapters from March 2020 to June 2024. Clinicians from these chapters were trained to refer caregivers of PLWD to the DCC program. Masters‐level care consultants from each chapter would reach out to the referred caregiver via telephone, conduct a care consultation, and send a summary of the care consultation to the referring provider. Research Specialists contacted referred caregivers to complete research surveys. Details of these processes are provided below.

#### 2.1.1 Collaboration With Health Systems

The Alzheimer's Association Health System Directors in Illinois, Greater Missouri, and Washington collaborated with the Association local Chapters to identify prospect health system accounts and conduct a relationship‐mapping exercise to identify appropriate entry points. Health systems were selected primarily based on existing relationships between Alzheimer's Association chapters and health systems that could be leveraged for DCC. These included partnerships established through grant‐funded initiatives, connections through the Knight Family Foundation, and utilizing research and clinical champions as entry points. Consideration was also given to ensure diversity among participating health systems, including representation from both rural and urban settings and a diverse range of patient populations. Health System Directors were Alzheimer's Association staff members that partnered closely with health systems and organizations across the country to help implement sustainable, evidence‐based approaches that supported timely diagnosis, person‐centered care, and improved outcomes for individuals living with dementia and their caregivers. By leveraging their relationships with local health systems and experience in working with the healthcare community, they served as strategic partners for implementation and quality improvement initiatives.

Using a consultative sales approach, the Health Systems Directors gained a better understanding of the health system needs in terms of providing quality care to PLWD and their caregivers. Based on the identified needs, the Health System Directors shared appropriate products, including the DCC Initiative, with the health system. Program adoption decisions were frequently made by a combination of senior clinical leadership and operational managers responsible for workflow redesign. The fact that DCC imposed no direct cost on the health system, required minimal workflow disruption, and provided specialized dementia expertise not available internally or as robustly were persuasive facilitators for adoption.

As a next step, the Health System Director shared the DCC Program Agreement with the health system account for review and feedback. The DCC Program Agreement clearly outlined the scope of the program and rules and responsibilities of each partner. The Alzheimer's Association program and legal team worked in collaboration with the health system to incorporate their feedback and make appropriate changes. Once the Program Agreement was finalized and signed by both parties, implementation planning activities commenced, including:

- Establishing referral workflow and assessing feasibility of integrating the referral form in partner's electronic health record (EHR)
- Identifying partner sites/clinics/hospitals for DCC implementation
- Conducting an orientation meeting with identified partner health system staff and leadership
- Ongoing stewardship and training to maintain and build referrals

### 2.2 Program Implementation

#### 2.2.1 Healthcare Provider Training

The Alzheimer's Association DCC outreach team delivered customized 20‐min training sessions to healthcare provider teams at each participating site. Training content was tailored to each site's referral workflow and typically covered: (1) DCC program overview and benefits for health systems, providers, patients, and caregivers; (2) referral procedures including consent processes, form completion, and submission protocols; and (3) case studies demonstrating DCC services for PLWD and caregivers. In the Alzheimer's Association training and promotional materials provided to healthcare providers, clinicians were asked to identify caregivers who may benefit from DCC. Examples of appropriate referrals included caregivers supporting individuals with memory concerns related to dementia, a new dementia diagnosis, or situations in which caregiver stress or PLWD care needs were increasing. Referrals were made at the discretion of the healthcare provider and with consent and interest from the caregiver.

#### 2.2.2 Flexible Referral Systems

The Association Health System Director collaborated with each site to establish convenient referral processes tailored to provider preferences:

- Electronic integration: DCC referral forms were embedded directly into health system EHRs with customized quick‐reference guides and targeted training as needed.
- Paper‐based options: Referral pads were provided to practices preferring traditional forms, with secure fax submission to the Alzheimer's Association.
- Digital alternatives: Fillable PDF forms were developed for providers preferring electronic completion with email submission.

#### 2.2.3 Care Consultant Preparation

DCC care consultants completed comprehensive training including: Alzheimer's Association standard Information and Referral and Care Consultation curricula; mandatory shadowing of 4–6 calls with Masters‐level care consultants from the Association's 24/7 Helpline with supervised DCC call observations for quality assurance; training on constituent documentation using the Association's Personify Constituent Relationship Management system; learning how to use the Community Resource Finder (CRF) and other community resource tools; and orientation to the customized DCC toolkit covering preparation, delivery, and documentation protocols. Introductory calls with the DCC Project Manager, data collection team, Health System Directors, and physician outreach teams provided consultants with comprehensive program understanding.

DCC care consultants were Masters‐level clinicians (counselors, social workers, or in a related field) who were staff from the respective chapters participating in the DCC program. Regular refresher training courses for the care consultants were conducted by the Alzheimer's Association Helpline Training team. Care consultants participated in monthly clinical supervision sessions, which included case discussions and mentoring with the Association's Clinical Director. Care consultation calls were not recorded due to data privacy concerns, so there was no formal fidelity monitoring in place. However, Research Specialists reviewed care consultation call notes and session summaries and reported any notes, flags, issues, or recurring concerns to the DCC project managers. The DCC project manager worked with the team to strategize and resolve problems.

#### 2.2.4 Care Coordination Process

During consultations, care consultants collaboratively developed individualized action plans with caregivers. Caregivers engaged in 1‐h care consultation and received the action plan verbally and in writing after the consultation. They were encouraged to reach out to the Alzheimer's Association (via the Helpline) if they wanted additional care consultations or had any further questions or concerns. If there were new issues or significant barriers to completing the action plan at the 1‐month (post care consultation) survey mark, the Research Specialist would offer one follow‐up care consultation with the DCC care consultant.

Care consultants provided structured summaries of the care consultation and action plan to referring providers. These summaries, adapted from successful models used by the Massachusetts chapter DCC pilot, included specific action steps, presenting issues, and a comprehensive checklist of topics addressed during consultations to ensure clear communication with healthcare teams. The summaries were integrated into patient EHRs.

#### 2.2.5 Evaluation Partnership

**FIGURE 1:**
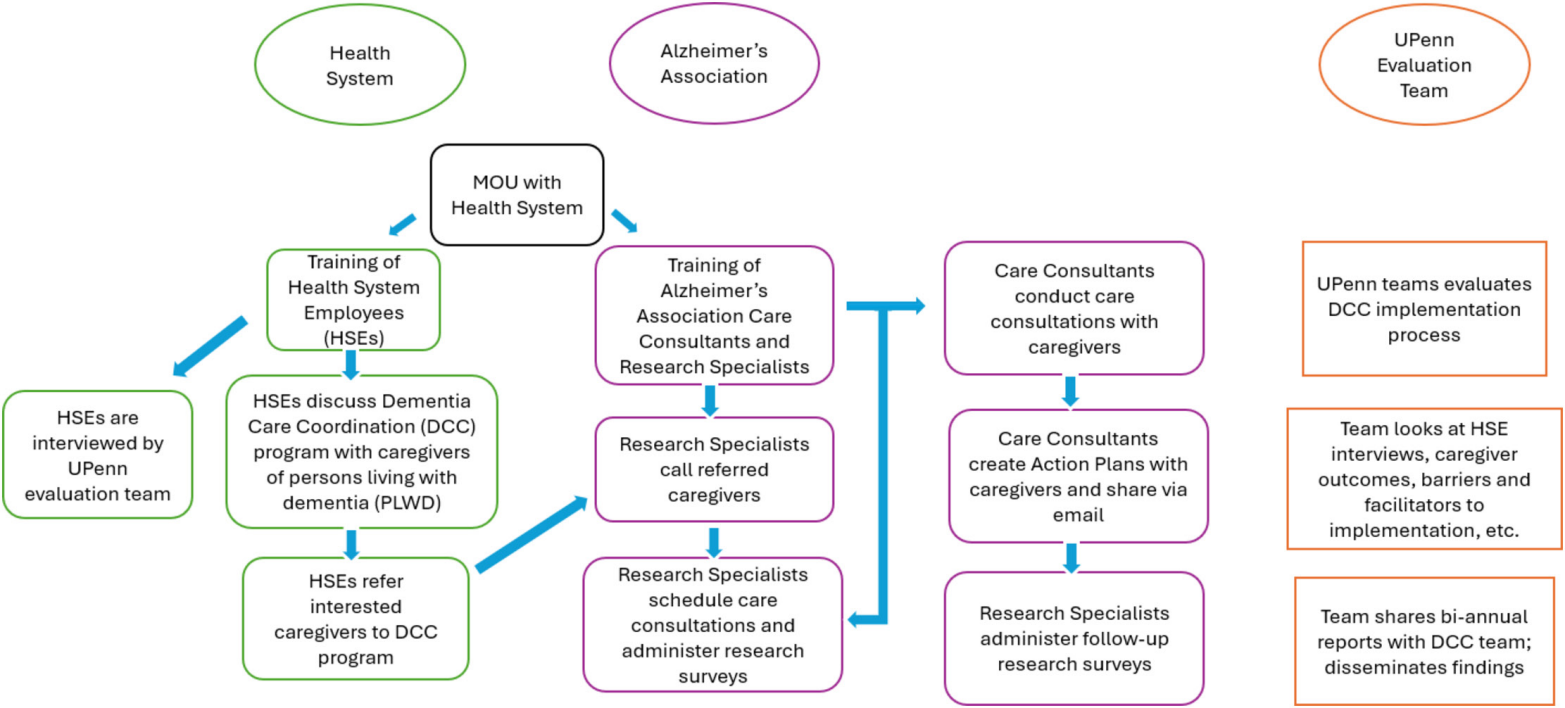
Overview of the dementia care coordination (DCC) process and evaluation. This figure provides an overview of the implementation of the DCC process. It describes the role of the health system, the Alzheimer's Association, and the University of Pennsylvania evaluation team.

The University of Pennsylvania School of Nursing evaluation team provided independent program assessment across all three chapters, collaborating with the Alzheimer's Association on research design, data analysis, reporting, and ongoing program improvement recommendations. Figure 1 provides an overview of the DCC process and evaluation. Evaluation report summaries were shared with implementation teams and health systems.

### 2.3 Data Collection

Research Specialists from the Alzheimer's Association conducted surveys with caregivers before and after their care consultations. The Alzheimer's Association research team specialists created a script to introduce the survey, invite caregivers to participate, and obtain verbal consent. Regular meetings were scheduled to discuss questions about data collection. Calls were recorded and reviewed for fidelity monitoring for the baseline and follow‐up surveys to evaluate if bias was introduced on the call. At the end of each quarter, the data was downloaded from the Constituent Relationship Manager (CRM) and reviewed for cleaning and missing data. Any personal identifying information was removed before sharing with the evaluation team for analysis. All data collection procedures and data sharing were approved by the University of Pennsylvania IRB (Protocol #834813).

### 2.4 Measures

#### 2.4.1 Caregiver and PLWD Assessment

Alzheimer's Association research team specialists collected baseline dyad demographics (gender, age, education, ethnicity, veteran status, caregiver role, PLWD living situation, and PLWD diagnosis) during initial phone contact following provider referral. The baseline survey included two validated instruments: the PROMIS Item Bank Self‐Efficacy for Managing Emotions Short Form 8A (PROMIS; measuring caregivers' confidence in handling their own emotional responses to caregiving) [4] and the general self‐efficacy scale (GSE; measuring caregivers' broader belief in their ability to handle challenging situations) [5]. Follow‐up surveys at 1‐ and 3‐months post‐consultation repeated these measures and added assessments of caregiver action plan implementation, caregiver barriers to action step completion, and self‐reported caregiver utilization of Alzheimer's Association services and resources (also referred to as resource utilization).

Additional program data captured: care consultation topics, delivery methods, and intensity levels; subsequent caregiver‐consultant contacts including frequency, dates, and follow‐up requests; referring provider health system affiliation and appointment context; and regional chapter assignment. Healthcare utilization questions regarding PLWD hospitalizations and emergency department visits were added during the final study months, assessed at baseline and 3‐month follow‐up.

#### 2.4.2 Health System Employee Assessment

Healthcare personnel completed surveys covering professional demographics (degree, specialty, and monthly dementia patient volume), referral practices (DCC referral frequency, methods, and ease of use), caregiver presenting needs, action plan summary utilization, and overall program satisfaction. Employees also completed Organizational Readiness for Implementing Change (ORIC) [6] surveys at program initiation and participated in qualitative interviews exploring DCC implementation barriers and facilitators (Document S2).

#### 2.4.3 Implementation Framework

The Consolidated Framework for Implementation Research (CFIR) [7] and Reach, Effectiveness, Adoption, Implementation, and Maintenance (RE‐AIM) [8] frameworks systematically guided measure development, data collection, and outcome analysis. CFIR domains informed the development of health system employee interview guides exploring intervention characteristics, outer setting factors, inner setting readiness, and implementation processes across diverse healthcare contexts. RE‐AIM components structured outcome evaluation by examining program reach across demographic groups, effectiveness through caregiver self‐efficacy measures, adoption patterns among healthcare providers, implementation fidelity through process metrics, and maintenance potential through sustainability assessments.

### 2.5 Statistical Analysis

#### 2.5.1 Descriptive and Comparative Analyses

Descriptive statistics characterized caregiver and PLWD demographics overall and by regional chapter, with between‐chapter comparisons using *F*‐tests for continuous variables and chi‐square tests for categorical variables. Caregiver PROMIS and GSE scores were compared across timepoints (baseline, 1, 3 months) using *t*‐tests, with subgroup analyses conducted for caregivers whose PLWD experienced emergency department visits or hospitalizations.

#### 2.5.2 Outcome Assessment

Three binary improvement indicators were created based on baseline to 3‐month changes in PROMIS total raw scores, PROMIS total t‐scores, and GSE total scores (1 = improved, 0 = no change/worsened). Bivariate logistic regression examined associations between score improvements and baseline participant characteristics. Probability proportion tests (Binomial and Sign tests) analyzed the likelihood of PROMIS and GSE score improvements from baseline to follow‐up periods.

#### 2.5.3 Process Evaluation

Frequency analyses characterized caregiver resource utilization, action plan uptake, and health system employee survey responses. Caregiver‐reported barriers to action implementation were qualitatively coded by two independent reviewers using NVivo to identify major themes. The two coders performed inductive coding, co‐developed a codebook, and engaged in discussions to resolve discrepancies. Thematic saturation was achieved.

All analyses used SAS 9.4 with significance set at *α* = 0.05.

## 3 Results

### 3.1 Participant Characteristics

The study enrolled 1865 unique participants, with 1810 from the three primary chapters (Table 1). Caregivers averaged 62.3 years of age (SD = 13.6), were predominantly female (73.5%), White (69.8%), and had some college education or higher (82.9%). Most were either children (48%) or spouses (39%) of PLWD, totaling 87% of the sample. PLWD averaged 78.5 years of age (SD = 8.6), were 62.4% female and 70.3% White (Table S1). The majority of PLWD lived with family (78.2%) and had an Alzheimer's disease or related dementia diagnosis (88.8%). Chapter distribution included: Illinois (*n* = 888), Greater Missouri (*n* = 455), Washington State (*n* = 467), and other chapters (*n* = 55).

**TABLE 1:** Caregiver sample descriptive statistics.

Characteristic	Overall (*N* = 1865)	Greater Missouri (*N* = 455)	Illinois (*N* = 888)	Washington State (*N* = 467)	Other (*N* = 55)	*p*
Caregiver age						< 0.0001
*n*	1855	452	884	464	55	
Mean (SD)	62.33 (13.56)	65.34 (12.79)	59.84 (13.40)	64.67 (13.69)	57.75 (12.56)	
Caregiver gender						0.0108
Male	484 (26.1%)	125 (27.8%)	201 (22.7%)	146 (31.4%)	12 (22.2%)	
Female	1364 (73.5%)	323 (71.8%)	682 (77.0%)	318 (68.4%)	41 (75.9%)	
Other	7 (0.4%)	2 (0.4%)	3 (0.3%)	1 (0.2%)	1 (1.9%)	
Caregiver highest education						< 0.0001
Less than high school degree	34 (1.8%)	8 (1.8%)	22 (2.5%)	4 (0.9%)	0	
High school graduate or equivalent	281 (15.2%)	97 (21.6%)	121 (13.7%)	58 (12.5%)	5 (9.3%)	
Some college	497 (26.8%)	135 (30.0%)	216 (24.4%)	134 (28.9%)	12 (22.2%)	
Bachelor's degree	597 (32.2%)	129 (28.7%)	287 (32.5%)	160 (34.5%)	21 (38.9%)	
Post/professional degree	443 (23.9%)	81 (18.0%)	238 (26.9%)	108 (23.3%)	16 (29.6%)	
Caregiver ethnicity						< 0.0001
White	1298 (69.8%)	429 (94.9%)	432 (48.7%)	394 (84.5%)	43 (78.2%)	
Black/African American	302 (16.2%)	12 (2.7%)	265 (29.9%)	19 (4.1%)	6 (10.9%)	
Other	260 (14.0%)	11 (2.4%)	190 (21.4%)	53 (11.4%)	6 (10.9%)	
Caregiver family role						< 0.0001
Child	894 (48.0%)	168 (37.1%)	518 (58.3%)	171 (36.6%)	37 (67.3%)	
Spouse	727 (39.0%)	223 (49.2%)	255 (28.7%)	241 (51.6%)	8 (14.5%)	
Other	242 (13.0%)	62 (13.7%)	115 (13.0%)	55 (11.8%)	10 (18.2%)	

*Note: p* values are based on *F* test for continuous variables and Chi‐square test for categorical variables. SD stands for standard deviation.

Survey completion rates were: baseline only (32%), partial follow‐up (28%), and complete three‐survey participation (40%). There were no significant differences in gender or education distribution among caregivers who completed only the baseline survey, completed one follow‐up survey, or completed all three surveys. White caregivers accounted for 63% of baseline‐only completers, 70% of partial follow‐up completers, and 75% of three‐survey completers. Among baseline‐only completers, 30% were spouses and 55% were children of PLWD. Among partial follow‐up completers, 39% were spouses and 49% were children of PLWD. Among three‐survey completers, 47% were spouses and 41% were children of PLWD. Compared with three‐survey completers, baseline‐only completers were, on average, 4 years younger, and partial follow‐up completers were 2 years younger. For ethnicity and caregiver role, effect sizes were small (all < 0.10). Differences in caregiver age were small to modest (effect sizes = 0.17 and 0.32).

### 3.2 Self‐Efficacy Outcomes

Caregiver self‐efficacy outcomes were mixed across follow‐up periods, with improvements and declines occurring in roughly equal proportions. Specifically, the PROMIS scale measures caregivers' confidence in managing their own emotional responses to caregiving (e.g., handling negative feelings, frustration, and worry), while the GSE reflects their general sense of capability in managing difficult life situations. At 1 month, 45% of caregivers improved on the PROMIS emotions management scale, 11% remained stable, and 44% worsened (Table 2). General self‐efficacy (GSE) scores showed a similar pattern: 41% improved, 17% remained stable, and 42% worsened. At 3 months, results were similar: PROMIS scores improved in 44%, remained stable in 14%, and worsened in 42%; GSE scores improved in 40%, remained stable in 14%, and worsened in 45% (Table 2). The near‐symmetrical distribution of improvement and worsening across both measures indicates that self‐efficacy scores were not consistently altered by program participation.

**TABLE 2:** Survey score proportion change.

Change	Frequency	Percent
PROMIS change from baseline to 1 month		
Worsened	471	44.1
No change	119	11.14
Improved	478	44.76
PROMIS change from baseline to 3 months		
Worsened	392	42.02
No change	126	13.5
Improved	415	44.48
GSE change from baseline to 1 month		
Worsened	446	41.8
No change	186	17.43
Improved	435	40.77
GSE change from baseline to 3 months		
Worsened	421	45.22
No change	134	14.39
Improved	376	40.39

*Note:* PROMIS stands for patient reported outcomes measurement information system. It is referring to the PROMIS Item Bank Self‐Efficacy for Managing Emotions Short Form 8A. GSE stands for the general self‐efficacy scale.

Mean scores on the PROMIS emotions management scale did not change significantly from baseline to one or 3 months (all *p* > 0.4). However, mean GSE scores declined slightly (0.44 points) from baseline to 3 months, a statistically significant though small difference (*p* = 0.001). Table 3 presents binomial probability tests asking a specific statistical question: Among caregivers whose scores changed in either direction, was the proportion who improved significantly greater than 50%? This test confirmed that, among those who changed, slightly more than half improved (all *p* < 0.05). However, this finding should be interpreted carefully given that approximately equal numbers improved and worsened in the full sample (Table 2), and the test excludes those whose scores did not change. Table 4 shows the probability of non‐decrease, or the likelihood that caregiver scores stayed the same or increased. In clinical terms, these statistical results do not indicate that the program reliably improved caregiver self‐efficacy; rather, they reflect that, among those who changed, improvement was marginally more common than worsening. No caregiver or care recipient demographic characteristics (age, gender, race, education, relationship to caregiver, etc.) were significantly associated with score changes across any measure (results not shown).

**TABLE 3:** Binomial probability test (improvement).

Binomial test	Percent	*p*
PROMIS baseline to 1‐month	44.76	< 0.001
PROMIS baseline to 1‐month (T score)	44.76	< 0.001
GSE baseline to 1‐month	40.77	< 0.001
PROMIS baseline to 3‐month	44.48	< 0.001
PROMIS baseline to 3‐month (T score)	44.48	< 0.001
GSE baseline to 3‐month	40.39	< 0.001

*Note:* PROMIS stands for patient reported outcomes measurement information system. It is referring to the PROMIS Item Bank Self‐Efficacy for Managing Emotions Short Form 8A. GSE stands for the general self‐efficacy scale.

**TABLE 4:** Binomial probability test (non‐decrease) as a sensitivity analysis.

Binomial test	Percent	*p*
PROMIS baseline to 1‐month	55.90	< 0.001
PROMIS baseline to 1‐month (T)	55.90	< 0.001
GSE baseline to 1‐month	58.20	< 0.001
PROMIS baseline to 3‐month	57.98	< 0.001
PROMIS baseline to 3‐month (T)	57.98	< 0.001
GSE baseline to 3‐month	54.78	0.0035

*Note:* PROMIS stands for patient reported outcomes measurement information system. It is referring to the PROMIS Item Bank Self‐Efficacy for Managing Emotions Short Form 8A. GSE stands for the general self‐efficacy scale.

### 3.3 Program Engagement and Resource Utilization

Caregivers demonstrated high engagement with DCC services and action planning. At 1 month, 63% completed action steps, with callback services (13%) and other services (15%) most frequently utilized. Callback services involved caregivers requesting additional contact with a care consultant after the initial care consultation. Planned future utilization included helpline services (22%) and support groups (24%). By 3 months, action step completion increased to 69%, with continued high use of callback (12%) and other services (14%), plus increased utilization of the Alzheimer's Association website, education programs, and support groups. Action plan understanding remained consistently high (90% at 1 month), with 71% following through on some (more than one) or all action steps at 3 months. Caregivers rated action plan helpfulness on average at 77/100 (1 month) and 75/100 (3 months).

Qualitative analysis of uptake barriers revealed four major themes: caregiver burden (time constraints, stress, multiple caregiving responsibilities, work‐life balance); low caregiver readiness; caregiver/PLWD medical issues; and PLWD resistance to services. Qualitative data was extracted from the sample described in Table 1.

### 3.4 Regional Chapter Variations

*Greater Missouri:* Caregivers were the oldest (mean age 65.3, SD = 12.8) with the highest proportion of White participants (94.9%) and lowest racial diversity. PLWD had the highest male representation (42.9%) and educational attainment patterns favoring high school completion. No significant changes in self‐efficacy scores were observed.

*Illinois:* This chapter demonstrated highest caregiver gender diversity (77% female) and racial diversity (48.7% White, 29.9% Black, and 21.4% other races). PLWD were the oldest (mean age 79.3, SD = 9.1) with similar diversity patterns. Self‐efficacy scores remained stable across timepoints.

*Washington State:* Washington had the lowest female caregiver representation (68.4%), highest spousal caregiving (51.6%), and youngest PLWD (mean age 77.4, SD = 8.7). Unlike other chapters, this group showed significant decreases in both PROMIS (0.56 decrease, *p* = 0.028) and GSE scores (0.52 decrease at 1 month, *p* = 0.027; 0.73 decrease at 3 months, *p* = 0.005).

### 3.5 Healthcare Utilization Impact

Among caregivers receiving DCC whose PLWD experienced emergency department visits or hospitalizations during the program, self‐efficacy patterns varied by timing and sites. At baseline, 151 caregivers reported recent PLWD hospitalizations (average stay 8.5 days, SD = 11.4), with no significant overall score changes but specific item decreases in handling negative feelings. At 3‐month follow‐up, 65 caregivers reported hospitalizations (average stay 8 days, SD = 14.7), showing significant PROMIS score decreases from 1 to 3 months (1.6‐point decrease, *p* = 0.016). Fourteen PLWD experienced hospitalizations at both timepoints, with this high‐utilization subgroup showing the largest caregiver self‐efficacy decreases (2.78‐point PROMIS decrease from 1 to 3 months, *p* = 0.02). In Greater Missouri, 27 (5.9%) PLWD had an ED visit or hospitalization. In Illinois and Washington State the corresponding numbers were 68 (7.7%) and 23 (4.9%) respectively.

### 3.6 Health System Employee Perspectives

ORIC surveys indicated strong organizational readiness for implementation despite COVID‐19 challenges. Survey respondents were primarily physicians (MDs/DOs including primary care physicians, neurologists, geriatricians, and more), nurses, and social workers, predominantly from Illinois (highest participating health systems), followed by Missouri and Washington State chapters. Employees found referral processes fairly easy, primarily using fax or EPIC systems, though they expressed preferences for more integrated workflows.

Employee satisfaction was high, with perceived patient satisfaction and willingness to recommend the program to peers. Key advantages identified included caregiver support and education, complex care coordination assistance, cost‐free implementation, time savings, and enhanced reach to underserved populations. Disadvantages centered on referral workflow inefficiencies, limited follow‐up communication, and occasional provider forgetfulness about the program.

### 3.7 Implementation Barriers and Facilitators

*Primary barriers included:* COVID‐19 pandemic impacts (reduced in‐person engagement, decreased patient visits, marketing challenges); healthcare employee time constraints and engagement difficulties; suboptimal referral form integration; caregiver attrition after referral; resource accessibility limitations; inconsistent action plan summary utilization; and high baseline caregiver burden affecting program engagement.

*Key facilitators included:* Strong health system outreach and relationship building; DCC team adaptability; cross‐chapter knowledge sharing; positive healthcare employee program reviews addressing care gaps; excellent caregiver testimonials; workflow integration feasibility; and strong health system champions driving referral rates.

**FIGURE 2:**
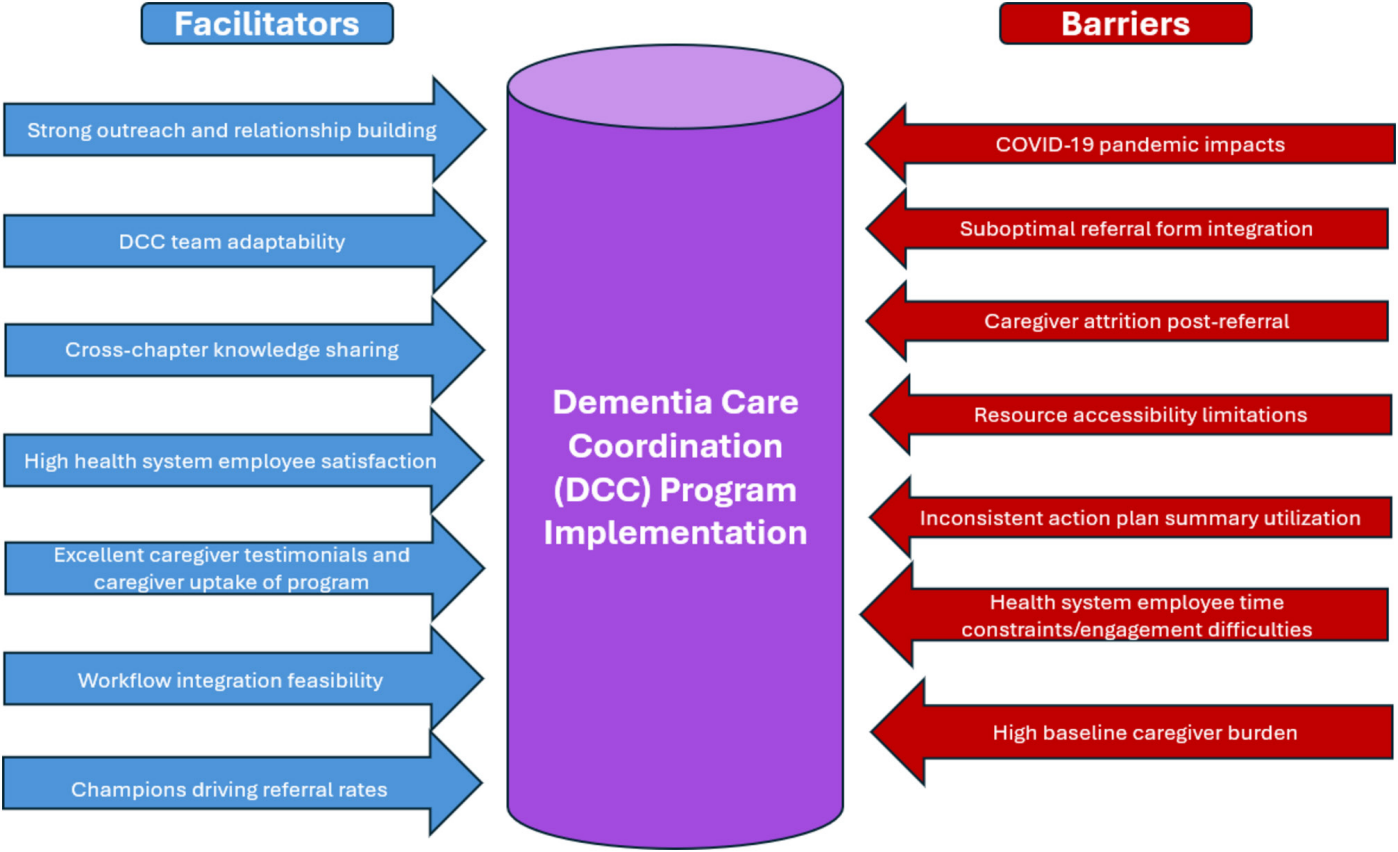
Summary of key implementation barriers and facilitators. This figure portrays the main barriers and facilitators to program implementation. These barriers and facilitators were identified by the evaluation team.

Figure 2 portrays a summary of the main barriers and facilitators.

## 4 Discussion

### 4.1 Program Effectiveness and Scalability

This multi‐state implementation of the DCC program demonstrated that a telephone‐based DCC model can be feasibly delivered across diverse healthcare systems serving more than 1800 caregivers. Notably, caregiver engagement with the program was strong: 90% understood their action plans, 70% implemented at least some action steps, and 80% utilized recommended community resources. This is meaningful given the well‐documented difficulty of engaging dementia caregivers who face competing demands. However, the program's impact on caregiver self‐efficacy, the primary caregiver outcome, was less clear. Roughly equal proportions of caregivers improved and worsened on both the emotions management and general self‐efficacy measures, without statistically significant mean change on either scale. These findings are important to consider in interpreting what the program achieved and for whom.

We had hypothesized that stable self‐efficacy scores might represent a meaningful outcome given the progressive nature of dementia caregiving stress and the tendency for caregiver emotional well‐being to decline over time. Several studies of structured caregiver interventions, including the Partners in Dementia Care program on which DCC was modeled, have reported prevention of worsening as a meaningful benchmark in this population [3]. However, a finding that roughly half of participants improved while roughly half worsened is difficult to attribute to the program itself, and raises the question of whether unmeasured factors, such as disease progression, life events, or baseline characteristics not captured here, are the primary drivers of these self‐efficacy trajectories. In particular, the subgroup of caregivers whose care recipients experienced repeated hospitalizations showed the steepest self‐efficacy declines, suggesting that medical crisis severity may overwhelm the program's support capacity for some families.

### 4.2 Healthcare System Integration and Provider Adoption

High healthcare provider satisfaction and perceived patient benefit validate the program's integration potential within existing clinical workflows. Providers recognized DCC as addressing critical care gaps while offering practical advantages: cost‐free implementation, time savings, and enhanced reach to underserved populations. However, implementation barriers, particularly around referral workflow optimization and provider engagement, highlight the importance of systematic integration strategies rather than add‐on approaches [9].

The regional variations observed, particularly Washington State's different outcome patterns, underscore the need for implementation flexibility while maintaining core program fidelity. Washington showed small but statistically significant declines in both PROMIS and GSE scores. Several contextual factors may explain this pattern: this chapter had a greater proportion of spousal caregivers, a group consistently found to experience greater emotional burden and faster increases in distress over time due to intensive day‐to‐day care. In addition, PLWD in Washington were younger on average, a characteristic that could be associated with more complex behavioral symptoms and earlier onset dementia. These findings suggest that successful scaling requires adaptive implementation approaches tailored to local healthcare contexts and populations while preserving evidence‐based program components.

### 4.3 Critical Implications for Medicare GUIDE Implementation

The DCC program's findings offer important insights at a time when the Medicare's Guiding an Improved Dementia Experience (GUIDE) Model implementation is currently launching nationally and with similar aims of providing comprehensive, coordinated dementia care. Several key parallels emerge:

*Proof of Concept:* DCC's telephone‐based coordination approach demonstrated feasibility and effectiveness across diverse healthcare systems. The high action plan uptake and resource utilization rates provide proof‐of‐concept for the care coordination model. The GUIDE model will test a more comprehensive model that follows the person and their caregivers across the course of the disease utilizing a dementia care navigator and clinical care team.

*Workflow Integration Lessons:* DCC implementation revealed that referral process optimization and EMR integration are critical for provider adoption. These insights of preference for integrated workflows over standalone systems suggest GUIDE programs may want to prioritize seamless clinical integration from implementation outset which aligns with other care coordination efforts [10].

*Caregiver Engagement Strategies:* The identification of time constraints and competing responsibilities as primary engagement barriers offers actionable intelligence for GUIDE programs designing caregiver support services. DCC's success in achieving high engagement despite these barriers demonstrates that well‐designed telephone coordination can overcome traditional participation obstacles.

*Quality Metrics and Evaluation*: DCC's comprehensive evaluation framework, utilizing validated self‐efficacy measures and implementation science approaches, provides a methodological template for GUIDE programs required to demonstrate effectiveness and inform ongoing program refinement.

### 4.4 Sustainability and Expansion Framework

The multi‐state implementation success, combined with strong provider satisfaction and caregiver outcomes, establishes a compelling evidence base for systematic program expansion. Key sustainability factors identified include: flexible referral mechanisms accommodating diverse provider preferences; robust care consultant training ensuring service quality; and ongoing implementation support addressing evolving barriers.

For Medicare GUIDE programs, these findings suggest that telephone‐based care coordination represents a viable, cost‐effective approach to meeting program requirements while achieving meaningful caregiver and clinical outcomes. The DCC model's demonstrated effectiveness across diverse populations and healthcare settings provides confidence that similar approaches can succeed within the GUIDE framework.

## 5 Future Directions and Policy Implications

### 5.1 Subgroup Considerations and Program Targeting

Because no demographic characteristics predicted self‐efficacy improvement, we cannot identify a clear profile of the caregiver most likely to benefit from DCC based on available data. However, clinical observations from the program suggest that caregivers who are newly referred, who have not yet accessed community resources, and whose care recipients are in earlier or more stable disease stages may derive the most benefit from a single‐contact consultation model. Conversely, caregivers managing acute or recurring medical crises, such as repeated hospitalizations, and those with high baseline burden may require more intensive or sustained support than a brief telephone coordination model provides. Future iterations of DCC or similar programs such as GUIDE should consider embedding stepped‐care pathways that direct higher‐burden caregivers to more intensive services, and prospectively track which caregiver‐care recipient dyads benefit most.

### 5.2 Limitations

Several important limitations of this evaluation must be acknowledged. First, this was an uncontrolled implementation study without a comparison group, so observed self‐efficacy trajectories cannot be attributed to DCC participation specifically; natural disease progression and external life events may explain much of the observed variability. Second, only 40% of enrolled caregivers completed all three surveys, potentially limiting the generalizability of longitudinal findings and introducing the possibility of attrition bias. We theorize that several factors likely contributed to a lower response rate: high caregiver burden and time constraints (identified in qualitative data); single contact intervention model and telephone‐based survey protocol made follow‐up reachability challenging; and the COVID‐19 pandemic potentially impacted caregiver capacity for follow‐up. No demographic differences were found between responders and non‐responders and given that most observed effect sizes were very small, we believe the likelihood of meaningful attrition bias is limited. Third, healthcare utilization data (emergency visits and hospitalizations) were added to the study in the later months, limiting the sample available for those analyses. Additionally, formal fidelity monitoring of care consultation content and delivery was lacking. However, all care consultants completed a standardized training and ongoing mentoring curriculum, and used uniform workflows with templated documentation, suggesting similar dosage and delivery structure across chapters. Finally, the absence of robust qualitative data from caregivers themselves about their program experience constrains our ability to interpret what elements, if any, were most helpful or effective. Future controlled trials are needed to establish whether self‐efficacy improvement, rather than stability, is achievable through telephone‐based coordination, and to identify which program elements drive caregiver benefit.

## 6 Summary

As healthcare systems nationwide prepare for GUIDE implementation, the DCC experience offers critical implementation guidance for establishing effective DCC programs. The evidence supports telephone‐based coordination as a scalable, effective model capable of achieving the care coordination goals that are a central component of GUIDE's success. Continued evaluation and program refinement, guided by implementation science frameworks, will ensure optimal outcomes for families affected by dementia while informing broader healthcare policy initiatives addressing this growing public health challenge.

The convergence of DCC evidence along with Medicare GUIDE implementation over the next 8 years represents a unique opportunity to translate research findings directly into healthcare policy and practice, potentially improving care coordination for millions of families nationally while establishing sustainable models for dementia care excellence.

## Author Contributions

N.A.H. developed the overall manuscript and evaluation design. L.H. contributed to statistical analyses and write‐up. S.T. assisted with manuscript development and content synthesis. S.L., S.F., K.F., L.S., and M.M. assisted with methods sections and overall manuscript edits.

## Funding

The University of Pennsylvania evaluation team was funded by the Alzheimer's Association.

## Conflicts of Interest

K.F., S.L., M.M., S.F., and L.S. are employees of the Alzheimer's Association. The remaining authors declare no conflicts of interest.

## Supporting information


**Table S1:** PLWD sample descriptive statistics. This table provides an overview of the characteristics of the persons living with dementia (PLWD) that the caregivers in this study provide care for.
**Document S2:** Health system employee interview guide. This document provides a list of questions that the evaluation team researchers asked health system employees during interviews.
