# Supplementary material for: Multi‐State Implementation of the Alzheimer's Association Dementia Care Coordination (DCC) Program
Source: J Am Geriatr Soc. 2026 Apr 30;74(8):2410–9. doi: 10.1111/jgs.70475 (PMC13496339; doi:10.1111/jgs.70475)
Supplement: Supplementary file 1 [file JGS-74-2410-s001.pdf]

Supplemental Table S1: PLWD Sample Descriptive Statistics

	Overall (N=1865)	Greater Missouri (N=455)	Illinois (N=888)	Washington State (N=467)	Other (N=55)	P-value
PLWD Age						0.0018
n	1839	449	873	462	55	
Mean (SD)	78.48 (8.63)	78.17 (7.57)	79.26 (9.09)	77.42 (8.71)	77.75 (7.61)	
PLWD Living Situation						<.0001
Alone	273 (14.7%)	63 (13.9%)	125 (14.1%)	64 (13.7%)	21 (38.2%)	
Living with Family	1453 (78.2%)	374 (82.4%)	688 (77.8%)	364 (78.1%)	27 (49.1%)	
Other	133 (7.2%)	17 (3.7%)	71 (8.0%)	38 (8.2%)	7 (12.7%)	
PLWD Diagnosis						<.0001
Alcohol-Related Dementia	7 (0.4%)	3 (0.7%)	2 (0.2%)	2 (0.4%)	0	
Alzheimer's Disease	762 (41.0%)	200 (44.2%)	358 (40.4%)	180 (38.6%)	24 (43.6%)	
Creutzfeld-Jakob	1 (0.1%)	0	1 (0.1%)	0	0	
Dementia Suspected	196 (10.5%)	36 (8.0%)	108 (12.2%)	44 (9.4%)	8 (14.5%)	
Dementia Unspecified	502 (27.0%)	107 (23.7%)	280 (31.6%)	106 (22.7%)	9 (16.4%)	
Frontotemporal/Pick's Dementia	41 (2.2%)	11 (2.4%)	14 (1.6%)	14 (3.0%)	2 (3.6%)	
Huntington’s	1 (0.1%)	0	0	1 (0.2%)	0	
Lewy Body Dementia	47 (2.5%)	15 (3.3%)	13 (1.5%)	19 (4.1%)	0	
Mild Cognitive Impairment	51 (2.7%)	15 (3.3%)	14 (1.6%)	20 (4.3%)	2 (3.6%)	

Mixed Dementia	99 (5.3%)	16 (3.5%)	31 (3.5%)	48 (10.3%)	4 (7.3%)	
No Diagnosis	1 (0.1%)	0	1 (0.1%)	0	0	
Other Dementia	15 (0.8%)	4 (0.9%)	5 (0.6%)	5 (1.1%)	1 (1.8%)	
Parkinson's Disease	20 (1.1%)	7 (1.5%)	9 (1.0%)	2 (0.4%)	2 (3.6%)	
Unknown	24 (1.3%)	3 (0.7%)	16 (1.8%)	5 (1.1%)	0	
Vascular Dementia	93 (5.0%)	35 (7.7%)	35 (3.9%)	20 (4.3%)	3 (5.5%)	
PLWD Gender						0.0006
Male	683 (36.8%)	193 (42.9%)	289 (32.7%)	188 (40.3%)	13 (24.1%)	
Female	1156 (62.4%)	256 (56.9%)	584 (66.1%)	275 (59.0%)	41 (75.9%)	
Other	15 (0.8%)	1 (0.2%)	11 (1.2%)	3 (0.6%)	0	
PLWD Ethnicity						<.0001
White	1306 (70.3%)	426 (94.5%)	429 (48.4%)	406 (87.1%)	45 (81.8%)	
Black/African American	307 (16.5%)	11 (2.4%)	269 (30.4%)	20 (4.3%)	7 (12.7%)	
Other	245 (13.2%)	14 (3.1%)	188 (21.2%)	40 (8.6%)	3 (5.5%)	
PLWD Highest Education						<.0001
Less than High School Degree	210 (11.6%)	27 (6.1%)	154 (17.9%)	24 (5.2%)	5 (9.1%)	
High School Graduate or Equivalent	546 (30.1%)	162 (36.7%)	245 (28.6%)	128 (27.9%)	11 (20.0%)	
Some College	414 (22.8%)	107 (24.2%)	167 (19.5%)	124 (27.1%)	16 (29.1%)	
Bachelor's degree	371 (20.5%)	92 (20.8%)	159 (18.5%)	110 (24.0%)	10 (18.2%)	

Post/Professional Degree	272 (15.0%)	54 (12.2%)	133 (15.5%)	72 (15.7%)	13 (23.6%)	

Notes:
P values are based on F test for continuous variables and Chi-square test for categorical variables. PLWD stands for person living with dementia.

Supplemental Document S2: Health System Employee Interview Guide

1. Are there, or have there been, programs similar to Dementia Care Coordination – which we will refer to as DCC – in your health system?
    a. How does the DCC program compare to treatment as usual or existing services in your health system for older adults diagnosed with dementia and their families?

This next question is a three-part question:

2. What are the potential advantages and disadvantages of the DCC program for the individuals with dementia and their families?
    a. What are the potential advantages and disadvantages of the DCC program for the health system internally?
    b. What are the potential advantages and disadvantages of the DCC program for you specifically in your role?

3. How well do you think the DCC program will meet the needs of individuals being diagnosed with dementia within your health system? In what ways?
   Prompts: Will it improve access to services, resource linkage, and care coordination?
   Will it personalize support for patients?
   Will it be perceived as useful?

    a. How well do you think it will meet the needs of their families?

   Prompts: Will it reduce stress or burden on caregivers and family?
   Will it improve access to services, resource linkage, and care coordination?
   Will it personalize support for caregivers and family?
   Will it be perceived as useful?
   Will it be perceived as flexible?

4. What barriers will individuals diagnosed with dementia and their families face in participating in DCC?

   Prompts: Willingness and motivation to participate?
   Understanding of the program (utility, etc.)?
   Emotional shock of diagnosis?
   Feel embarrassed, like a burden, etc.?

   a. How do you suggest eliminating potential barriers?

5. What aspects of the DCC program facilitate participation and motivate use? (and this can be providers/participants)

6. Do you have any suggestions for making the DCC program most useful for older adults diagnosed with dementia in your setting within the health system?

7. Do you think the DCC program gives your health system a competitive advantage over other organizations that provide dementia care and services?

   Ask prompt: Would something about the program bring more patients to your organization, instead of another one in your area?

8. How do you think your health system's culture (define culture) affects the implementation of the DCC program?

   Culture definition: general beliefs/practices, patient interaction, what your health system prioritizes

   a. Can you describe a time that the health system tried to implement a new program or practice and how culture factored into that?

9. Do you expect to have sufficient resources to implement and administer the DCC program?
    a. **[If yes]:**
        i. What resources are you counting on?
        ii. What resources have you received so far?
        iii. What resources would you have liked to receive when DCC was first being implemented?
        iv. Are there any resources that you would like to receive in the future?
        v. How easy is it to access the resources (that you currently have)?
    b. **[If no]:**
        i. What resources would you need and why are they not available to you?

10. (You don't have to name names here) Who do you go to or who do you ask if you have questions about the DCC program or its implementation?
    a. How available are these individuals?
    b. How comfortable are you with reaching out to these individuals?

11. How confident are you that you will be able to implement and sustain the DCC program within the scope of your role?
    a. What gives you that level of confidence?
    b. How has COVID-19 changed this, if at all?

12. How confident are you that the health system will be able to implement and sustain the DCC program?
    a. What gives you that level of confidence (or lack of confidence)?
    b. How has COVID-19 changed this, if at all?
    c. What is currently measured or tracked in order to understand whether the program is being used effectively?
    d. What is currently measured or tracked for quality improvement?

13. Do you have any suggestions for improvement of the DCC program at this time or any final thoughts or questions you'd like to share with us?

14. Thank you again for taking the time to talk with us. We really appreciate your time and feedback.
